# Early prediction of gestational diabetes mellitus using maternal demographic and clinical risk factors

**DOI:** 10.1186/s13104-024-06758-z

**Published:** 2024-04-15

**Authors:** Yanqi Wu, Paul Hamelmann, Myrthe van der Ven, Sima Asvadi, M. Beatrijs van der Hout-van der Jagt, S. Guid Oei, Massimo Mischi, Jan Bergmans, Xi Long

**Affiliations:** 1 Department of Electrical Engineering, Eindhoven University of Technology, Eindhoven, The Netherlands; 2 Philips Research, Eindhoven, The Netherlands; 3 Department of Biomedical Engineering, Eindhoven University of Technology, Eindhoven, The Netherlands; 4 Department of Obstetrics and Gynaecology, Máxima Medical Center, Veldhoven, The Netherlands

**Keywords:** Gestational diabetes mellitus, Early prediction, Model validation, Maternal demographics, Clinical risk factors

## Abstract

**Objective:**

To build and validate an early risk prediction model for gestational diabetes mellitus (GDM) based on first-trimester electronic medical records including maternal demographic and clinical risk factors.

**Methods:**

To develop and validate a GDM prediction model, two datasets were used in this retrospective study. One included data of 14,015 pregnant women from Máxima Medical Center (MMC) in the Netherlands. The other was from an open-source database nuMoM2b including data of 10,038 nulliparous pregnant women, collected in the USA. Widely used maternal demographic and clinical risk factors were considered for modeling. A GDM prediction model based on elastic net logistic regression was trained from a subset of the MMC data. Internal validation was performed on the remaining MMC data to evaluate the model performance. For external validation, the prediction model was tested on an external test set from the nuMoM2b dataset.

**Results:**

An area under the receiver-operating-characteristic curve (AUC) of 0.81 was achieved for early prediction of GDM on the MMC test data, comparable to the performance reported in previous studies. While the performance markedly decreased to an AUC of 0.69 when testing the MMC-based model on the external nuMoM2b test data, close to the performance trained and tested on the nuMoM2b dataset only (AUC = 0.70).

**Supplementary Information:**

The online version contains supplementary material available at 10.1186/s13104-024-06758-z.

## Introduction

It is estimated that approximately 1 in 7 pregnant women develops gestational diabetes mellitus (GDM) during pregnancy [1]. Pregnant women with diagnosed GDM might require medication to control their blood sugar level. An uncontrolled level of blood glucose during pregnancy might contribute to large birth weight, preterm birth, pre-eclampsia, respiratory distress syndrome, jaundice, hypoglycemia, and stillbirth. In addition, GDM patients have an up to 87% risk of developing type 2 diabetes in 5–10 years after their delivery [2, 3]. The consequences of GDM for babies include, for example, an abnormally high birth weight and hypoglycemia after birth [4]. Various studies have demonstrated that early lifestyle modifications during pregnancy can have an effect in reducing the risk of developing GDM [5]. By making lifestyle adjustments (such as improving diet and physical activity) as early as possible in pregnancy, typically before week 15, and maintaining them throughout the pregnancy, this effect is enhanced [6]. Hence, to facilitate effective treatment and lifestyle adjustments, it is pivotal to accurately predict the risk of developing GDM early in pregnancy.

In the past decade, dozens of studies have been reported in the field of early risk stratification or prediction of GDM using electronic medical records (EMRs) before its diagnosis [7, 8]. We summarized 22 EMR-based GDM prediction studies published since 2010 in the Supplementary Materials. The prediction performance, measured by the area under the receiver-operating-characteristic curve (AUC), ranged from 0.57 to 0.95 [7–10]. Those studies included data from different cohorts with, for example, different sample size and GDM prevalence. Moreover, the risk factors used for GDM prediction were different between studies. The most frequently used risk factors were body mass index (BMI), age, race (or ethnicity), parity, gravidity, family history of diabetes, and history of GDM. Although some studies considered biomarkers and demonstrated their good predictability in early prediction of GDM [11, 12], many of those biomarkers are either not routinely measured or unavailable in the datasets used in our work.

In this work, we aimed at developing an early GDM prediction model based on the widely used maternal demographic and clinical risk factors available in the first trimester. We first performed internal validation on an in-house dataset and then validated the model on an external open-source dataset.

## Materials and methods

### Datasets

Two datasets were included in this retrospective study for model development and (internal and external) validation for GDM prediction.

The first dataset was an in-house dataset, called “MMC dataset”, containing data from pregnant women who visited the Máxima Medical Center (MMC), Veldhoven, the Netherlands, and gave birth between January 2012 and December 2017. The study received a waiver for ethical approval from the medical ethical committee of MMC. The inclusion criteria for the MMC dataset were pregnant women who delivered at MMC and had related obstetrical records, aged between 18 and 45 years, and without diagnosed type I or type II diabetes before pregnancy, i.e. pre-existing diabetes. In addition, for modelling, samples with missing data, either risk factors or GDM diagnosis, were excluded or imputed. A total of 15,709 samples from 14,015 pregnant women were analyzed in our study.

The second dataset was obtained from an open-source database called “Nulliparous Pregnancy Outcomes Study: Monitoring Mothers-to-Be” (nuMoM2b) [13]. In the nuMoM2b study, 10,038 nulliparous women with singleton pregnancies were recruited from hospitals affiliated with eight clinical centers in the USA. They were recruited if they had a viable singleton gestation and were between 6 and 14 weeks of gestation. The detailed GDM diagnosis criteria in the nuMoM2b study were described by Haas et al. [13]. The exclusion criteria for the nuMoM2b dataset were pregnant women with an age < 13 years, a history of three or more pregnancy losses, donor oocyte pregnancy, planned pregnancy termination, pre-existing diabetes, malformations likely to be lethal and aneuploidies known at or before enrolment, and inability to provide informed consent [13]. This led to a total of 8,720 pregnant women who were included in our study.

### Risk factors

As stated, the most frequently used risk factors from the first trimester were considered for modelling. They were maternal demographics including age, BMI, and ethnicity, as well as clinical risk factors including parity, gravidity, family history of diabetes, and history of GDM. These risk factors are often readily available from the hospital EMR system, as they can more easily be collected during the first trimester of pregnancies compared to other variables such as biomarkers requiring a blood test or ultrasound-related records needing an ultrasound scan.

### Prediction modelling

The seven risk factors were considered machine learning features for early prediction of GDM. Given the simplicity and good interpretability of logistic regression (LR), it has been the most widely used algorithm in EMR-based GDM prediction [14], which motivated us to employ LR in our study. Elastic net regularization was applied in LR modelling to cope with potential collinearity and overfitting issues, where several parameters were required to be optimized such as regularization strength C, penalty L1/L2, and class weight.

In general, for machine learning, a dataset should be divided into three subsets: training, validation, and test sets [15]. The training set is used for model training, and the validation set is used for parameter optimization of the trained model. The test set is considered a hold-out set, used only for model evaluation to avoid bias. Considering both the MMC and the nuMoM2b datasets are highly imbalanced, simple random splitting could lead to significant deviations in the fractions of positive samples between subsets, which may in turn leads to model distortion. Stratified split is a widely used method for imbalanced dataset to reduce sample bias. Because in the MMC dataset, some pregnant women had multiple birth records, it was crucial to ensure that all the records from same pregnant woman were always kept in the same set. Therefore, we used an “individual-level” stratified split on the MMC dataset. First, all individuals (pregnant women) were divided into two groups based on whether they had any delivery record diagnosed as GDM. Then a stratified method was performed on both groups of individuals to split the dataset into MMC-training (60%), MMC-validation (20%) and MMC-test (20%) sets. The prediction performance (AUC) was computed for both nulliparous and multiparous pregnancies of the MMC-test set. In the nuMoM2b dataset, all participants only had one delivery record. The stratified split method was performed in terms of GDM diagnosis to divide the entire dataset into nuMoM2b-training (60%), nuMoM2b-validation (20%) and nuMoM2b-test (20%) sets. This ensured that same or similar percentages of samples for both GDM and non-GDM were assigned into the three subsets. For external validation, the LR model was trained and optimized on the MMC-training and MMC-validation sets, while tested on the nuMoM2b-test set. Because the nuMoM2b cohort included only nulliparous pregnancies, parity, and history of GDM were set to zero. To examine the generalizability of the MMC-based model to the nuMoM2b dataset, we performed a comparison validation that trained and optimized an LR predictor on the nuMoM2b-training and nuMoM2b-validation sets, and tested on the nuMoM2b-test set.

To understand the feature contribution to the GDM prediction, feature coefficients of the LR models trained based on the MMC-training and the nuMoM2b-traning data were provided, where a higher absolute coefficient means a stronger contribution to the model. In addition, the odds ratio for each risk factor was also calculated to evaluate its correlation with GDM.

## Results

The demographic and clinical risk factors in the MMC dataset and the nuMoM2b dataset are presented in Table 1.

**Table 1:** Demographic and clinical risk factors of pregnant women. Values are presented as mean ± standard deviation, percentage, or number (percentage)

	MMC dataset (*N* = 15,837 from 14,015 pregnant women)		nuMoM2b dataset (*N* = 8720 from 8720 pregnant women)	
Outcome	GDM (*N* = 641, 4%)	Non-GDM (*N* = 15,196, 96%)	GDM (*N* = 376, 4%)	Non-GDM (*N* = 8344, 96%)
Pre-pregnancy BMI^#^	27.83 ± 6.06**	24.31 ± 4.95	33.88 ± 7.58**	29.91 ± 6.27
Age	31.85 ± 4.82**	30.22 ± 4.51	29.54 ± 5.82**	26.81 ± 5.61
Ethnicity - Non-Hispanic White/Black - Hispanic/Mediterranean - Asian - Rest Ethnicity	493 (76.9%)** 39 (6.1%) 58 (9.0%) 51 (8.0%)	12,656 (83.3%) 617 (4.1%) 562 (3.7%) 1361 (8.9%)	205 (54.5%)* 101 (26.9%) 41 (10.9%) 20 (5.3%)	5110 (61.2%) 2511 (30.1%) 382 (4.6%) 341 (4.1%)
Parity	0: 277 (43.2%)** 1: 251 (39.1%) 2: 76 (11.8%) >=3: 37 (5.8%)	0: 8120 (53.4%) 1: 4971 (32.7%) 2: 1501 (9.9%) >=3: 604 (4.0%)	NA	NA
Gravidity	1: 231 (36.0%)** 2: 216 (33.7%) >=3: 194 (30.3%)	1: 6891 (45.3%) 2: 4339 (28.5%) >=3: 3966 (27.2%)	1: 272 (72.3%) 2: 79 (21.0%) >=3: 25 (6.6%)	1: 6217 (74.5%) 2: 1575 (18.9%) >=3: 549 (6.6%)
Family history of diabetes	49.4%**	17.1%	35.1%**	20.0%
History of GDM	26.2%**	1.0%	NA	NA

NA: not applicable due to the inclusion of only nulliparous pregnancies in the nuMoM2b dataset

*p < 0.05,

**p < 0.001 between the GDM and non-GDM group

^#^For the nuMoM2b data, the pre-pregnancy BMI was confirmed through self-reported 3 months before pregnancy weight. For the MMC data, the pre-pregnancy BMI was confirmed through the self-reported non-pregnancy weight, but the specific time corresponding to this weight was not available

The detailed statistics of the demographic and clinical risk factors for the subsets after data split (including MMC-training, MMC-validation, MMC-test, nuMoM2b-training, nuMoM2b-validation, and nuMoM2b-test) were described in the Supplementary Materials.

**Fig. 1:**
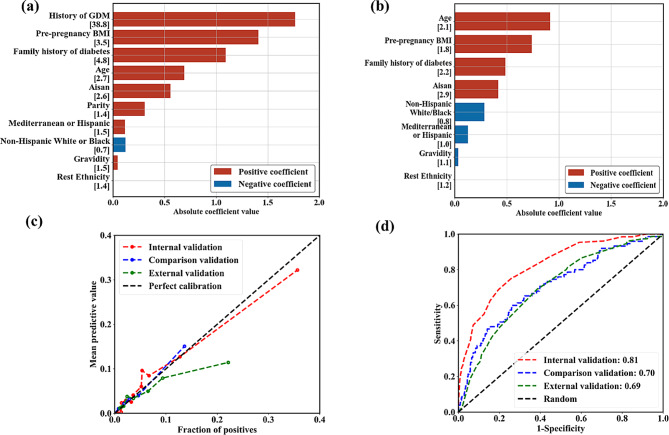
**a**: Feature coefficient in the internal validation model. **b**: Feature coefficient in the comparison validation model. **c**: Calibration curve for internal, comparison, and external validation models. **d**: AUC curve for internal, comparison and external validation models. Bar colour in plot **a** and **b** represents the sign of the coefficient, where red indicates positive correlation with GDM and blue means negative correlation. Odds ratio between each feature and GDM was described in the rectangular brackets after the feature’s name. The odds ratio of Age, BMI, Parity, and Gravidity was calculated between Age > = 25 and GDM, between BMI > = 25 and GDM, between parity number > 0 and GDM, and between gravity number > 1 and GDM, respectively. Colour of the dash-dot curves in plot **c** and **d** represents different models, including internal validation model (red), comparison validation models (blue), and external validation model (green)

The performance of early GDM prediction using different datasets for training and testing is presented in Table 2. The internal validation showed an AUC of 0.81, indicating an 81% probability that a randomly selected patient with GDM would receive a higher risk score than whom without GDM in the MMC dataset. The model for external validation had a decreased AUC of 0.69, comparable to that obtained using the comparison model that was trained, validated, and tested on the nuMoM2b dataset (AUC = 0.70). The AUC and calibration curves for internal, external and comparison validation are plotted in Fig. 1.

**Table 2:** Summary of early GDM prediction performance (evaluated by AUC) using different datasets for training and testing

Training data	Test data	Nulliparous/multiparous (test data)	Test AUC
MMC^a^	MMC^a^	All	0.81
		Nulliparous	0.75
		Multiparous	0.83
MMC^b^	nuMoM2b^b^	Nulliparous	0.69
nuMoM2b^c^	nuMoM2b^c^	Nulliparous	0.70

^a^ Internal validation: 60%, 20%, and 20% of the MMC data for training, validation, and testing, respectively

^b^ External validation: 60% and 20% of the MMC data for training and validation, respectively, and 20% of the nuMoM2b dataset for testing

^c^ Comparison validation: 60% 20%, and 20% of the nuMoM2b data for training, validation, and testing, respectively

In Fig. 1a and b, the absolute value of each bar represents the contribution of the feature in the model. For the internal validation model, ‘history of GDM’ had the highest contribution to the model and the highest odds ratio associated with GDM. The odds ratio for ‘history of GDM’ is 38.8, indicating that pregnant women who had GDM before are 38.8 times more likely to have GDM in a following pregnancy than those who never had GDM before. For the comparison validation model, the feature ‘Age’ had the largest contribution to the model, while ‘history of GDM’ and ‘parity’ had no contribution since they were not available in the nuMoM2b dataset. To evaluate the stability of the models, mean and standard deviation as well as 95% confidence interval (CI) of AUC results for the internal, external and comparison validations were obtained after running 100 times with different stratified (random) splits of training, validation, and test sets, as reported in the Supplementary Materials. The results showed a relatively small standard deviation and range of 95% CI for almost all models.

From the calibration plot, unlike the external model, the curves for the internal and the comparison model seemed to follow the perfect calibration curve relatively well. However, for the internal validation, the highest fraction of positives in the MMC-test dataset (including both nulliparous and multiparous pregnancies) was about 0.36. The highest fraction of positives for the nulliparous pregnancies in the MMC-test set was less than 0.2, close to that in the nuMoM2b-test set with only nulliparous pregnancies.

## Discussion

In this study, we developed and validated models for GDM prediction using routinely collected risk factors that are available during or before the first trimester, and the prediction results could help provide timely medical intervention and promote early lifestyle changes to reduce the risk of developing GDM. In the internal validation, a major finding is that the GDM risk prediction for the nulliparous pregnancies was much more difficult than that for multiparous pregnancies, evidenced by the model performance measured by AUC (0.75 versus 0.83). This could be partially explained by the inexistence of pregnancy history in nulliparas. Actually, the overall contribution of pregnancy history in the GDM risk prediction model can be as high as 40% as reported by Artzi et al. [8], which corroborates our finding. In addition, we found that the external validation result for GDM prediction was clearly lower than the internal validation result (AUC of 0.69 versus 0.75 for nulliparous pregnancies). This indicates that the model trained from the MMC cohort might not generalize well to another cohort (nuMoM2b) having a different distribution in some important risk factors. For example, there existed clear discrepancies in age, BMI, and family history of diabetes between the two datasets, and these factors were highly ranked with respect to their contribution to the prediction models as shown in Table 1.

The calibration plot shows that the internal model and the comparison model seemed well calibrated. However, the external validation model that trained on the MMC-training set tended to overestimate the risk of GDM in the nuMoM2b-test set, particularly for women with a higher GDM risk where the predicted risk was higher than the observed risk. This could be due to the differences in the association of the risk factors with GDM for different cohorts. For example, in the MMC dataset, the probability of pregnant women having family history of diabetes who eventually developed GDM was 49%, which was higher than that in the nuMoM2b dataset (35%). As shown in Fig. 1, the risk factor ‘family history of diabetes’ was top ranked in the LR models for both internal and comparison validations. In addition, in the MMC dataset, the probability of GDM in the Mediterranean/Hispanic population was higher than the average. However, this was the opposite in the nuMoM2b dataset, which would likely cause the probability provided by the model in external validation to be higher than the actual probability. Donovan et al. [16] also reported that the model trained on nulliparous pregnant women in a California dataset overestimated the risk of pregnant women in a dataset from Iowa.

To maximize the model’s interpretability and reproducibility, this study selected LR as the algorithm for GDM prediction. As shown in the Supplementary Materials, LR showed similar results in predicting GDM compared with the other algorithms for internal, external, and comparison validations. Nonetheless, more advanced algorithms should be evaluated when including larger datasets with more risk factors in future work.

It is important to note that, both datasets are highly imbalanced with a minority class accounting for less than 5% of the total samples per dataset, leading to difficulty in predicting GDM as the minority class, in particular when the GDM samples are insufficient to represent the entire population of GDM patients. It is worth mentioning that the ethnicity categories defined in both datasets used in this study were different. To diminish the effect caused by such difference, we harmonized the categories for both datasets in order to make them comparable, as shown in Table 1. Even though, we observed that, the ethnicity of nearly half of the pregnancies in the nuMoM2b dataset was American Black, while the dominant ethnicity in the MMC dataset was European White. In addition, unlike the MMC dataset collected in the Netherlands including both nulliparous and multiparous pregnant women, the nuMoM2b dataset includes only nulliparous pregnancies in the United States.

### Limitations

The current study had several limitations. First, many often used risk factors that have demonstrated good predictive value such as glucose tolerant test, blood pressure, smoking history, polycystic ovary syndrome, daily exercise, and biomarkers, were not considered during modelling since these variables were not available in at least one of the datasets used in this study. Including more independent risk factors is therefore expected to further improve GDM prediction [17–20]. Second, the MMC and nuMoM2b datasets had different GDM diagnosis criteria as well as inclusion criteria, which would lead to bad model generalizability from one to the other dataset, regardless of the differences seen in some risk factors. Third, in both datasets, self-reported weight before pregnancy was used, where the specific time of the weight was unknown. For the MMC dataset, actual measurement of BMI before pregnancy or during the first trimester of pregnancy was not always available and for many pregnant women, their first BMI measurement was done after 20 weeks of gestation. These would lead to inaccuracy in training a GDM prediction model.

### Electronic supplementary material

Below is the link to the electronic supplementary material.

Supplementary Material 1

## Data Availability

Data from the nuMoM2b database are currently publicly available through the Eunice Kennedy Shriver National Institute of Child Health and Human Development (NICHD), National Institutes of Health Data and Specimen Hub (DASH; https://dash.nichd.nih.gov/). Data from the MMC database were collected in the Máxima Medical Center, Veldhoven, The Netherlands. Due to privacy regulations, the MMC data are not publicly available. Please contact Xi Long (x.long@tue.nl) for further information.

## Abbreviations

AUC: Area Under the Curve
EMR: Electrical Medical Record
GDM: Gestational Diabetes Mellitus
MMC: Máxima Medical Center
nuMoM2b: Nulliparous Pregnancy Outcomes Study: Monitoring Mothers-to-Be
BMI: Body Mass Index
CI: Confidence Interval
LR: Logistic Regression
