# Supplementary material for: Early prediction of gestational diabetes mellitus using maternal demographic and clinical risk factors
Source: BMC Res Notes. 2024 Apr 15;17:105. doi: 10.1186/s13104-024-06758-z (PMC11021008; doi:10.1186/s13104-024-06758-z)
Supplement: Supplementary file 1 — Supplementary Material 1 [file 13104_2024_6758_MOESM1_ESM.docx]

**GDM diagnosis standard in the Maxima Medical Center (MMC)**

Ealy-pregnancy (<15 weeks of gestation) Fasting Plasma Glucose (FPG) test and mid-pregnancy (24 to 28 weeks of gestation) 2 hours 75g-Oral Glucose Tolerance Test (OGTT) were used for the GDM diagnosis on pregnant women without pre-existed diabetes in MMC.

FPG test:
This test measures blood glucose levels before 15 weeks of gestation after an overnight fasting, typically for at least 8 hours. If the venous blood glucose > 7.1 mmol/l in FPG test, the pregnant women would be diagnosed s GDM.

2h 75g-OGTT:
The OGTT involves fasting overnight, followed by drinking a liquid containing a 75g glucose between 24 to 28 weeks of gestation. Venous blood samples would be taken before drinking, 1 hour after drinking and 2 hours after drinking. If the any of the following three criteria was satisfied during the OGTT that the pregnant women would be diagnosed as GDM:

- Fasting venous blood glucose > 5.1 mmol/l
- Venous blood glucose > 10 mmol/l 1 hour after drinking the glucose
- Venous blood glucose > 8.5 mmol/l 2 hours after drinking the glucose.

Only 20 in 641 pregnant women were diagnosed as GDM by FPG test and we estimated that this part of women did not have a large impact on the model’s prediction. For the pregnant women who did not meet the GDM determination conditions in FPG test or inexistent in FPG result, their OGTT results would be used to determine whether they have GDM.

**Descriptive statistics of risk factors**

**Table S1**. Descriptive statistics of demographic and clinical risk factors of pregnant women used for GDM prediction in different subsets of the MMC dataset (including training, validation, and test sets). Values are presented as number or mean ± standard deviation.

| Subset | **MMC-training set**  (60%) | **MMC-validation set**  (20%) | **MMC-test set**  (20%) |
| --- | --- | --- | --- |
| All Record number | 9483 | 3158 | 3196 |
| GDM record number | 381 | 129 | 131 |
| Age | 30.65 ± 4.54 | 30.79 ± 4.48 | 30.62 ± 4.46 |
| Pre-pregnancy BMI^#^ | 24.40 ± 4.96 | 24.46 ± 4.96 | 24.59 ± 5.47 |
| Ethnicity   - Non-Hispanic White/Black - Hispanic/ Mediterranean - Asian - Rest Ethnicity | 7892  372  376  843 | 2626  143  127  262 | 2631  141  126  298 |
| Parity | 0: 4721  1: 3139  2: 949  >=3: 674 | 0: 1608  1: 1036  2: 290  >=3: 124 | 0: 1577  1: 1047  2: 338  >=3: 119 |
| Gravidity | 1: 4296  2: 2745  >=3: 2442 | 1: 1477  2: 881  >=3: 703 | 1: 1383  2: 929  >=3: 781 |
| Family history of diabetes | 1738 | 573 | 598 |
| History of GDM | 190 | 55 | 61 |

NA: not applicable due to the inclusion of only nulliparous pregnancies in the nuMoM2b dataset.
^#^In MMC, the pre-pregnancy BMI was confirmed through the self-reported non-pregnancy weight, but the specific time corresponding to this weight was not available.

**Table S2**. Descriptive statistics of demographic and clinical risk factors of pregnant women used for GDM prediction in different subsets of the nuMoM2b dataset (including training, validation, and test sets). Values are presented as number or mean ± standard deviation.

|  | **nuMoM2b-training set** (60%) | **nuMoM2b-validation set** (20%) | **nuMoM2b-test set**  (20%) |
| --- | --- | --- | --- |
| **All Record number** | 5232 | 1745 | 1743 |
| **GDM record number** | 226 | 75 | 75 |
| **Age** | 26.95 ± 5.64 | 26.87 ± 5.70 | 26.85 ± 5.58 |
| **Pre-pregnancy BMI^#^** | 25.48 ± 6.33 | 25.44 ± 6.28 | 25.19 ± 5.80 |
| **Ethnicity**   - **Non-Hispanic White/Black** - **Hispanic/ Mediterranean** - **Asian** - **Rest Ethnicity** | 3205  1557  240  230 | 1054  516  76  99 | 1056  548  73  66 |
| **Parity** | NA | NA | NA |
| **Gravidity** | 1: 3895  2: 1012  >=3: 325 | 1: 1283  2: 331  >=3: 131 | 1: 1311  2: 314  >=3: 138 |
| **Family history of diabetes** | 1093 | 347 | 373 |
| **History of GDM** | NA | NA | NA |

NA: not applicable due to the inclusion of only nulliparous pregnancies in the nuMoM2b dataset.
^#^The pre-pregnancy BMI in nuMoM2b was confirmed through self-reported pregnancy weight three months before pregnancy.

**Determination of specific pregnancy time**

The nuMoM2b study is a well-designed cohort study. Pregnant women's data were collected through several visits during the pregnancy (6-13 weeks for visit 1, 15-21 weeks for visit 2, 25-31 weeks for visit 3) as well as the delivery visit. The specific expected start date of pregnancy and the detailed data collection date of each risk factor were all described in the nuMoM2b dataset as the difference compared to the date of delivery (https://dash.nichd.nih.gov/study/226675).

Compared with the nuMoM2b dataset, the MMC dataset was directly derived from the electronic medical records data of the hospital’s data lake. The start time and end time of a pregnancy were confirmed by the last menstrual period date and the delivery data in the records, respectively.

**Performance comparison between prediction algorithms**

**Table S3**. Mean ± standard deviation of AUC (95% confidence interval) for internal, external and comparison validations obtained from 100 times of stratified (random) split of data.

| **Algorithm** | **Internal validation** | **External validation** | **Comparison validation** |
| --- | --- | --- | --- |
| **Logistic Regression (LR)** | 0.808 ± 0.017  (0.805-0.811) | 0.694 ± 0.025  (0.689-0.699) | 0.710 ± 0.025  (0.705-0.715) |
| **Support vector machines (SVM)** | 0.810 ± 0.018  (0.806-0.814) | 0.694 ± 0.026  (0.689-0.699) | 0.698 ± 0.027  (0.693-0.703) |
| **Extreme gradient boosting (XGBoost)** | 0.815 ± 0.017  (0.812-0.818) | 0.707 ± 0.026  (0.702-0.712) | 0.706 ± 0.027  (0.701-0.711) |
| **Neural networks (NN)** | 0.806 ± 0.020  (0.802-0.810) | 0.681 ± 0.032  (0.675-0.687) | 0.697 ± 0.034  (0.690-0.704) |
